# Sleep Traits to the Risk of Breast Cancer Disease Incidence, Adverse Progression and Mortality: Evidence From a Global Systematic Review and Meta-Analysis

**DOI:** 10.3389/ijph.2025.1608535

**Published:** 2025-07-15

**Authors:** Jingya Zhang, Yongbo Lu, Ning Zhang, Wei Ning, Bin Zhu, Ying Mao

**Affiliations:** ^1^ School of Public Policy and Administration, Xi’an Jiaotong University, Xi’an, China; ^2^ Vanke School of Public Health, Tsinghua University, Beijing, China; ^3^ School of Public Health and Emergency Management, Southern University of Science and Technology, Shenzhen, China; ^4^ SUSTech Homeostatic Medicine Institute, School of Medicine, Southern University of Science and Technology, Shenzhen, China

**Keywords:** breast cancer, sleep, incidence, adverse progression, mortality

## Abstract

**Objectives:**

This study aimed to identify the effect of sleep traits on the risk of breast cancer incidence and adverse progression and mortality.

**Methods:**

Cohort studies measuring the relationship between sleep traits (including sleep quality and sleep duration) and breast cancer risk were eligible for inclusion. We searched the Web of Science, PubMed, EMBASE and Cochrane library databases for studies published between 2014 and 2024. Maximum covariate-adjusted odds ratio (OR) was combined. A fixed or a randomized effect model was applied according to the heterogeneity.

**Results:**

34 studies met the inclusion and exclusion criteria. Low quality sleep significantly increased the risk of incidence (OR:1.09, 95%CI:1.05–1.13), adverse progression (OR:1.55,95%CI:1.51–1.59), and specific mortality (OR:1.54, 95%CI:1.50–1.58) of breast cancer. Sleep duration >9 h had a poor effect on breast cancer-specific mortality (OR:1.45,95%CI:1.02–2.04).

**Conclusions:**

The available evidence points to sleep traits as primarily influencing progression in breast cancer patients and having a relatively small effect on breast cancer incidence. Prolonged sleep may lead to breast cancer-specific mortality, but more research is needed in the future to continue to explore the impact of sleep duration and breast cancer risk.

## Introduction

Breast cancer has emerged as the most prevalent malignancy among women globally, imposing a substantial public health burden worldwide. Particularly in some developing countries, breast cancer has led to an enormous case of incidence and mortality. According to Global Cancer 2022, breast cancer accounted for 2,296,840 new cases and 666,103 deaths among women worldwide, representing 23.76% of all female cancer cases and 15.44% of cancer-related deaths. These proportions were even higher in Africa, reaching 29.23% and 21.89% respectively [1]. This trend has prompted increasing attention to identifying modifiable risk factors that could potentially influence both the incidence and progression of breast cancer [2].

In recent years, there has been growing scientific interest in understanding how lifestyle-related factors contribute to breast cancer development and outcomes [3, 4]. While multiple lifestyle factors including diet, physical activity, and stress management have been extensively studied, sleep has emerged as a particularly compelling area of investigation due to its unique biological significance and potential for intervention [5]. Unlike other lifestyle factors that may require substantial behavioral changes or resources, sleep patterns are modifiable through relatively accessible interventions, making them specifically relevant from a public health perspective [6, 7].

Moreover, sleep represents a fundamental biological process that occurs daily for approximately one-third of human life, providing consistent and prolonged exposure that could significantly impact cancer-related biological pathways [8]. This ubiquitous process is directly involved in multiple critical biological systems known to influence carcinogenesis: the circadian rhythm regulatory network that controls DNA repair timing, the immune surveillance system that eliminates abnormal cells, and the hormonal axis that regulates growth factors and inflammatory responses [9, 10].

The biological plausibility for sleep-cancer associations is further strengthened by mounting experimental evidence. At the molecular level, sleep deprivation disrupts circadian clock genes, which regulate cell cycle checkpoints and DNA damage response pathways. This disruption impairs the nocturnal peak of DNA repair enzymes, allowing accumulation of mutations in oncogenes and tumor suppressor genes [11]. This DNA repair mechanisms is critical for preventing malignant transformations. Furthermore, in breast tissue specifically, sleep disruption suppresses melatonin production, which normally inhibits breast cancer cell growth through MT1 and MT2 receptors and suppresses aromatase-mediated estrogen synthesis. The resulting melatonin deficiency promotes breast cancer development by removing these anti-proliferative constraints and increasing local estrogen exposure, particularly relevant for hormone-sensitive breast cancers [12]. At the cellular level, sleep deprivation reduces natural killer cell cytotoxicity by up to 70% and decreases T-cell proliferation, compromising immune surveillance against malignant cells [13]. Studies demonstrate that sleep fragmentation promotes breast cancer metastasis by increasing tumor-associated macrophage infiltration and enhancing epithelial-mesenchymal transition through special signaling pathways. Additionally, sleep loss increases pro-inflammatory cytokines and angiogenic factors, creating a tumor-promoting microenvironment that facilitates cancer cell survival, proliferation, and metastatic spread [14].

The relationship between sleep characteristics and breast cancer has been examined in various epidemiological studies [15, 16]. These investigations have explored different aspects of sleep, including duration, quality, timing, and sleep disorders, in relation to breast cancer risk and prognosis [17, 18]. First, insufficient sleep is significantly related to an increased incidence of breast cancer [19], with studies documenting a 41% increased risk among women with extended exposure to rotating night shifts [20]. Beyond initial cancer development, sleep patterns may also impact disease progression and outcomes. Studies have demonstrated that chronic insomnia is associated with a 52% increased risk of metastatic progression among breast cancer patients, independent of other known prognostic factors [21]. This increased metastatic risk likely stems from sleep-disruption-induced physiological changes, including altered immune function and inflammatory responses. Sleep duration represents another critical dimension of sleep characteristics, potentially involving mechanisms of altered melatonin production, disrupted DNA repair processes, and elevated inflammatory markers [22]. Multiple cohort studies have demonstrated that both short and long sleep traits are associated with increased risk of breast cancer incidence and adverse progression (including tumor stage advancement, disease recurrence, and treatment-related complications) and mortality [18, 23, 24]. A longitudinal study of 2,456 early-stage breast cancer patients has found that those with poor sleep quality (PSQI >5) have 2.1-fold increased risk of progressing from stage I-II to stage III-IV within 3 years, compared to good sleepers [25]. Sleep disruption also predicts disease recurrence and complications, treatment tolerance, and other adverse events during active therapy [18]. Also, sleep duration showed clear associations with survival: both short and long sleep were associated with increased breast cancer-specific mortality respectively, independent of initial stage and treatment modality [24]. These findings suggest that sleep disturbances may impact both the initiation and progression of breast cancer through multiple mechanisms.

Several meta-analyses have attempted to synthesize this evidence with important contributions. Research examining sleep-disordered breathing has found significant associations with increased breast cancer risk [26]. Meta-analyses focusing on sleep duration reveal a potential U-shaped relationship in which both short and long sleep durations are associated with elevated breast cancer risk, though with notable heterogeneity across populations. However, existing studies present somewhat inconsistent findings [15, 27], and the dose-response relationship between sleep duration and cancer risk remains understood [28]. Similarly, conflicting results have been observed regarding the impact of sleep quality on breast cancer prognosis [29]. Moreover, existing systematic reviews have primarily focused on isolated sleep parameters (such as duration or specific sleep disorders) without comprehensively evaluating the full spectrum of sleep characteristics and their potential interactions. While individual studies have investigated specific sleep parameters or populations [30, 31], there is a notable absence of integrative reviews that systematically analyze how multiple sleep traits collectively influence both breast cancer incidence and progression across diverse populations.

To address this knowledge gap, we conducted a systematic review and meta-analysis to comprehensively evaluate the association between sleep traits and breast cancer risk. Specifically, based on our aim to validate the effect of sleep characteristics on the overall disease process in breast cancer, and considering the existing literature characteristics, this study aims to identify how sleep quality and sleep duration influence the risk of breast cancer incidence, adverse progression (tumor stage progression, recurrence, and complications) and mortality. By synthesizing evidence from global research, this review seeks to provide clearer insights into the role of sleep characteristics in breast cancer, potentially informing both preventive strategies and clinical management approaches [32, 33].

## Methods

### Inclusion and Exclusion Criteria

We included case studies and cohort studies with the following characteristics: (i) reported on breast cancer incidence or breast cancer adverse progression or breast cancer mortality; (ii) measured at least one sleep trait (sleep quality or sleep duration). We excluded studies that were published in languages other than English, those for which full text was not available, or that consisted of narrative reviews, comments or letters.

### Information Sources

**FIGURE 1:**
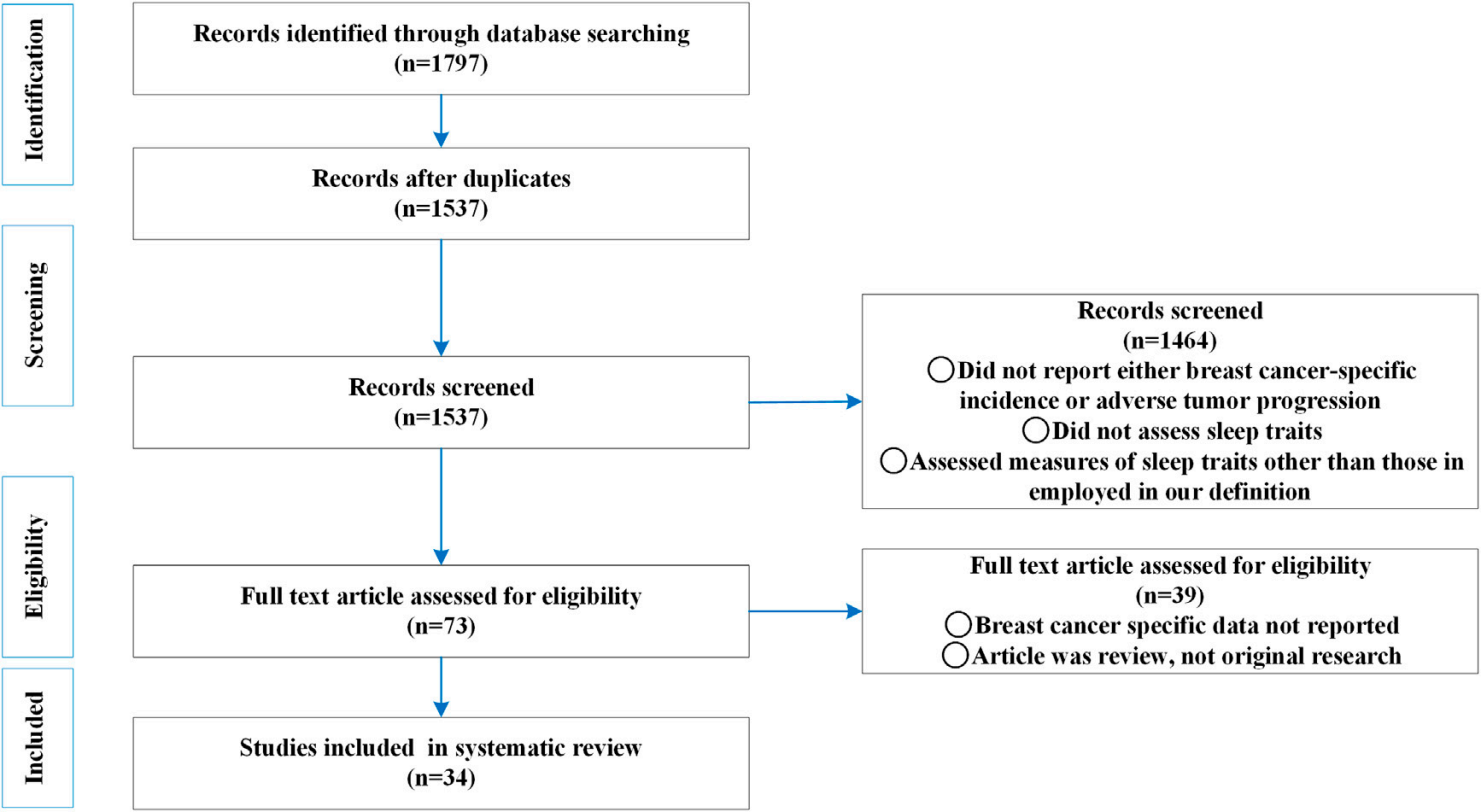
Preferred Reporting Items for Systematic Reviews and Meta-Analyses Flow diagram of literature search for the association between sleep traits and the risk of breast cancer incidence, adverse progression and mortality (Global, 2025).

We followed the PRISMA (Preferred Reporting Items for Systematic Reviews and Meta-Analyses) guidelines [Transparent Report of Systematic Reviews and Meta-Analysis (Moher et al. 2009)] as a methodological template for this review (Figure 1). Four databases (PubMed, EMBASE, Web of Science and Cochrane library) were searched to identify papers examining the association between sleep traits and the risk of breast cancer disease incidence, adverse progression and mortality. The effects of low quality sleep, sleep duration <6 h and sleep duration >9 h on the breast cancer risk were extracted. Low quality sleep includes terms such as sleep difficulties, insomnia, and sleep disorders. And measurement tools used include such as the Pittsburgh Sleep Quality Index, polysomnography, and self-report measures. The detailed search strategies are included in the Supplementary Material (Supplementary Table S1–S4). PubMed searches were conducted using a combination of Medical Subject Headings (MeSHs) and Keywords (in title or abstract.) EMBASE was searched using EMTREE and title/abstract search. Web of Science and Cochrane library were searched using keywords. The evolving approach to breast cancer control is demonstrated by the high frequency of updates to clinical practice guidelines [34], where older studies may no longer reflect current best practices or technical standards [35]. The role of lifestyle factors, including sleep, on breast cancer-related health indicators can be influenced by changes in clinical treatment modalities [36], creating new treatment-lifestyle synergies or antagonisms [37], and there is a particular need to dynamically assess the amount of lifestyle factor effects in different treatment contexts [38]. At the same time, we consider that studies in the last decade have typically adopted more rigorous study designs, statistical methods, and reporting standards, with higher quality literature [39]. Ultimately, database searching was carried out from 2014 to 2024.

### Screening and Selection Process

Two independent co-authors (JY and YB) screened titles and abstracts and then screened the full text of the identified articles against predefined eligibility criteria. Our search retrieved a total of 3,225 articles from PubMed, EMBASE, Web of Science and Cochrane library. After manually removing 1,688 duplicates, we were left with a total of 1,537 unique references. Of the 1,537 screened articles, 1,464 studies - that did not report sleep traits, reported measures of sleep traits other than those included in our definition, and did not report either incidence or progression were excluded. We obtained full text for all 73 remaining articles, and after review, we additionally excluded 39 articles that did not report breast cancer-specific outcomes or were themselves systematic reviews. The remaining 34 articles were included in this review. Author disagreements were resolved through discussion.

### Risk of Bias in Individual Studies

The risk of bias in the studies included in the review was rated independently by Y.B. and J.Y., following the same consensus procedure employed for study selection. The Newcastle-Ottawa Scale (NOS) quality assessment tool [40] was used for assessing risk of bias. A “star system” has been developed in which a study is judged on three broad perspectives: the selection of the study groups; the comparability of the groups; and the ascertainment of either the exposure or outcome of interest for case-control or cohort studies respectively. Studies with a score of 6 or higher were considered as high-quality research [21]. A summary figure of the assessed bias of the included studies was created. See Supplementary Material (Supplementary Table S5). We employed funnel plots to assess the potential bias of publication included. (Supplementary Figure S1)

### Data Analysis

Data were independently extracted by three co-authors (Y.B and J.Y), capturing Author/Year, Study Type and Period, Sample Amount, Outcomes, Sleep Traits, odds ratio or rate ratio or hazard ratio (OR/RR/HR) indicator, corresponding confidence intervals (CI), confounding factors adjusted and main conclusion. See Supplementary Material (Supplementary Table S6). OR, RR and HR are clinically and statistically similar in that they are all “relative effect sizes,” reflecting the risk of the exposure/intervention group compared with the control group, and, although the mathematical definitions differ, OR ≈ RR ≈ HR at low rates of outcome events (<10%) [41]. Also, it is possible to combine different effect sizes when study clinical homogeneity is high (e.g., similar interventions, populations, outcome definitions) [42]. Therefore, we harmonized and merged the three effect sizes. When multiple measures of association were reported, we reported results from the fully adjusted model. A fixed or randomized effect model was applied according to the heterogeneity. Forest plots [43] are used to display the results of the merge. Publication bias was assessed by funnel plots. All statistical analyses were carried out in Review Manager v.5.3 (Copenhagen: The Nordic Cochrane Centre, The Cochrane Collaboration 2014).

## Result

Following a systematic search and screening process, a total of 34 studies meeting the eligibility criteria were included in this review. Among these studies, 23 investigated the relationship between sleep quality or duration and breast cancer incidence, while 11 examined the association between sleep quality or duration and adverse prognosis in breast cancer patients. Geographically, the included studies predominantly originated from North America and Asia, with 14 studies (41.2%) from the United States, 9 studies (26.5%) from China, and 5 studies (14.7%) from the United Kingdom. The remaining studies were conducted in Norway (n = 1), France (n = 1), South Korea (n = 1), and Germany (n = 1), with one additional case-control study encompassing multiple Asian populations, respectively (Table 1).

**TABLE 1:** Characteristics of studies included for research of Sleep Traits to the Risk of Breast Cancer Disease Incidence, Adverse Progression and Mortality (Global, 2025).

Author/Year	Study type and period	Sample amount	Outcomes	Sleep traits	OR/HR/RR	Country
Zhang et al. [44] 2024	Prospective cohort 2006–2022	360,271 BC = 7,817	Breast cancer incidence	Sleep quality	HR 1.12 (1.05–1.20)	The United Kingdom
Yang et al. [45] 2019	Case–control 2013–2016	Case = 401 Control = 401	Breast cancer incidence	Sleep quality Sleep duration (<6 h)	OR 1.08 (0.90–1.87) OR 0.98 (0.82–1.95)	China
White et al. [46] 2017	Prospective cohort 2003–2014	50,884 BC = 2,736	Breast cancer incidence	Difficulty sleeping Sleep duration (<6 h) Sleep duration (>9 h)	HR 1.07 (0.93, 1.24) HR 0.94 (0.85, 1.04) HR 1.00 (0.86, 1.17)	The United States
Von et al. [47] 2024	Prospective cohort 2012–2019	39,555 BC = 1,085	Breast cancer diagnosis	Sleep quality Sleep duration (<5 h) Sleep duration (5–6 h) Sleep duration (>9 h)	HR 1.02 (0.84, 1.23) HR 1.08 (0.74, 1.59) HR 1.02 (0.86, 1.21) HR 0.99 (0.73, 1.33)	The United States
Sen et al. [48] 2017	Prospective cohort 1995–2012	33,332 BC = 862	Breast cancer incidence	Sleep quality	HR 2.38 (1.11, 5.09)	Norway
Richmond et al. [49] 2019	Case–control 2006–2016	149,005 BC = 2,740	Breast cancer incidence	Sleep quality	HR 1.02 (0.97, 1.08)	The United Kingdom
Liu et al. [50] 2023	Case–control 2008–2020	63,018 BC = 700	Breast cancer incidence	Sleep satisfaction Sleep duration (>9 h)	OR 1.17 (1.07, 1.29) OR 1.16 (0.92, 1.47)	China
Liu et al. [51] 2021	retrospective cohort 2000–2013	232,018 BC = 2,280	Breast cancer incidence	Sleep quality	HR 1.16 (1.07, 1.27)	Taiwan, China
Justeau et al. [52] 2020	Prospective cohort 2007–2017	8,748 BC = 67	Breast cancer incidence	Sleep quality	HR 1.14 (0.50–2.58)	French
Hurley et al. [53] 2020	Case–control 2012–2015	BC = 2,856 Control = 38,649	Breast cancer incidence	Sleep quality Sleep duration (<5 h) Sleep duration (>9 h)	OR 1.35 (0.99, 1.85) OR 1.06 (0.83, 1.36) OR 1.22 (1.02, 1.46)	California
Gao et al. [54] 2020	Case–control NR	BC = 1,200 Control = 1,200	Breast cancer incidence	Sleep quality	OR 1.33 (1.13, 1.56)	Asia
Feng et al. [55] 2024	Case–control NR	BC = 133,384 Control = 113,789	Breast cancer incidence	Sleep quality	OR 0.75 (0.49, 1.15)	The United Kingdom
Choi et al. [56] 2019	Prospective cohort 2007–2014	OSA = 45,699 227 Control = 228,502 955	Breast cancer incidence	Sleep quality	HR 1.20 (1.04–1.39)	Korea
Chang et al. [57] 2014	Prospective cohort 1997–2010	SA = 846 12 Control = 4,230 32	Breast cancer incidence	sleep apnea	HR 2.09 (1.06–4.12)	Taiwan, China
Qian et al. [58] 2015	Prospective cohort 1973–1989	40,013 BC = 1846	Breast cancer incidence	Sleep duration (<6 h) Sleep duration (>9 h)	RR 0.87 (0.64, 1.18) RR 1.00 (0.84, 1.19)	The United States
Ren et al. [59] 2014	Case–control 2010–2012	BC = 712 Control = 742	Breast cancer incidence	Sleep duration (<6 h) Sleep duration (>9 h)	OR 1.62 (1.18–2.24) OR 1.55 (1.14–2.10)	China
Shen et al. [23] 2019	Prospective cohort 2001–2018	10,802 BC = 429	Breast cancer incidence	Sleep duration (<6 h) Sleep duration (>9 h)	HR 1.71 (0.92–3.18) HR 1.38 (0.69–2.74)	Mexican merican
Shigesato et al. [60] 2020	Prospective cohort 1993–2013	74,481 BC = 5,790	Breast cancer incidence	Sleep duration (<6 h) Sleep duration (>9 h)	HR 1.03 (0.97–1.09) HR 1.05 (0.95–1.15)	The United States
Turner et al. [61] 2022	Case–control 2008–2013	BC = 1,543 Control = 1,560	Breast cancer incidence	Sleep duration (<6 h) Sleep duration (>9 h)	OR 0.92 (0.72–1.18) OR 1.01 (0.76–1.33)	Spain
Wang et al. [62] 2015	Case–control 2010–2012	BC = 652 Control = 669	Breast cancer incidence	Sleep duration (<6 h) Sleep duration (>9 h)	OR 1.53 (1.10–2.12) OR 1.59 (1.17–2.17)	China
Wong et al. [63] 2021	Prospective cohort 1999–2017	713,150 BC = 36,173	Breast cancer incidence	Sleep duration (<6 h) Sleep duration (>9 h)	RR 1.01 (0.95–1.07) RR 1.03 (0.95–1.12)	The United Kingdom
Xiao et al. [64] 2016	Case–control 2002–2009	BC = 518 Control = 42,435	Breast cancer incidence	Sleep duration (<6 h) Sleep duration (>9 h)	OR 1.04 (0.79, 1.36) OR 1.07 (0.80, 1.43)	The United States
Cai et al. [65] 2024	Case–control 2009–2024	BC = 263 Control = 1,526	Breast cancer incidence	Sleep duration (>9 h)	OR 1.05 (0.95,1.15)	The United States
Zhu et al. [66] 2018	Prospective cohort 2006–2018	4,219 Cases = 672	Breast cancer progression Breast cancer mortality	Sleep quality	HR 0.93 (0.69, 1.26) HR 1.04 (0.82, 1.31)	China
Vin et al. [67] 2018	Prospective cohort 2007–2017	84,424 Cases = 5,176	Breast cancer progression Breast cancer mortality	Sleep quality	OR 1.58 (1.29, 1.34) OR 0.72 (0.45, 1.14)	The United States
Soucise et al. [68] 2017	Prospective cohort 1994–2013	4,171 Cases = 320	Breast cancer progression	Sleep quality Sleep duration (<5 h) Sleep duration (>9 h)	OR 0.85 (0.68–1.07) OR 1.06 (0.76–1.49) OR 0.92 (0.65–1.30)	The United States
Liang et al. [18] 2019	Prospective cohort 2008–2017	1,580 Cases = 111	Breast cancer progression	Sleep quality Sleep duration (<6 h) Sleep duration (>9 h)	HR 3.08 (1.74,5.47) HR 1.45 (0.83,2.54) HR 2.33 (1.01,5.42)	China
Jacob et al. [69] 2018	Case-control 2000–2010	Cases = 5,706 Control = 5,706	Breast cancer progression	Sleep quality	OR 1.31 (1.20–1.44)	Germany
Chen et al. [70] 2022	Retrospective cohort 2009–2019	2,966 Cases = 488	Breast cancer mortality	Sleep quality	HR 1.51 (1.19–1.91)	Taiwan, China
Bach et al. [71] 2021	Retrospective cohort 2008–2017	6,656 Cases = 461	Breast cancer mortality	Sleep quality	HR 1.39 (1.04–1.87)	The United Kingdom
Marinac et al. [25] 2017	Prospective cohort 1995–2010	3,047 Cases = 1,114	Breast cancer progression Breast cancer mortality	Sleep duration <6 h (recurrence) Sleep duration >9 h (recurrence) Sleep duration <6 h (mortality) Sleep duration > 9 h (mortality)	HR 0.93 (0.77, 1.12) HR 1.48 (1.01, 2.00) HR 0.83 (0.67, 1.04) HR 1.52 (1.09, 2.13)	The United States
Trudel et al. [24] 2017	Prospective cohort 1976–2008	3,682 Cases = 412	Breast cancer mortality	Sleep duration <6 h Sleep duration >9 h	HR 1.13 (0.86–1.48) HR 1.46 (1.02–2.07)	The United States
Palesh et al. [31] 2013	Prospective cohort 2002–2012	97 Cases = 55	Breast cancer mortality	sleep duration	HR 0.99 (0.97–1.00)	The United States
Nair et al. [72] 2024	Prospective cohort 1996–2018	817 Cases = 132	Breast cancer mortality	sleep duration <5 h sleep duration >9 h	HR 0.71 (0.39–1.30) HR 1.16 (0.53–2.52)	The United States

Note: NR, not reported in the original study.

### Analysis of Included Articles

The included studies consisted of 12 cohort studies and 11 case-control studies investigating breast cancer incidence risk, and 10 cohort studies and 1 case-control study examining prognostic outcomes. All cohort investigations (n = 22) established follow-up periods extending beyond 1 year, providing sufficient timeframes for observing relevant breast cancer outcomes (Table 1). The confounding factors primarily controlled for in these studies included demographic factors (race, age, income, education, reproductive age); health status (BMI, depression, family history of breast cancer, history of other cancers); and risk behaviors (smoking, alcohol consumption, diet, medication use, physical activity). All included studies were assessed to be of high quality using Newcastle-Ottawa Scale (NOS) quality assessment tools (Supplementary Table S5). Inspection of the funnel plot demonstrated no substantial evidence of publication bias in our meta-analysis. (Supplementary Figure S1) These contemporary studies, with their scientific designs and diverse populations, provide a robust foundation for evaluating the impact of sleep quality on breast cancer incidence and prognostic progression. Among the literature included, fifteen studies (65.22%) indicated that sleep quality and duration are associated with increased risk of breast cancer incidence, whereas eight studies concluded evidence did not support the relationship. In terms of adverse progression, four studies (66.67%) suggested that sleep quality and duration are correlated with breast cancer progression; four studies (50%) demonstrated that compromised sleep quality and duration are associated with elevated breast cancer mortality rates (Supplementary Table S6).

### Analysis of Sleep Traits Effects on Breast Cancer Incidence

**FIGURE 2:**
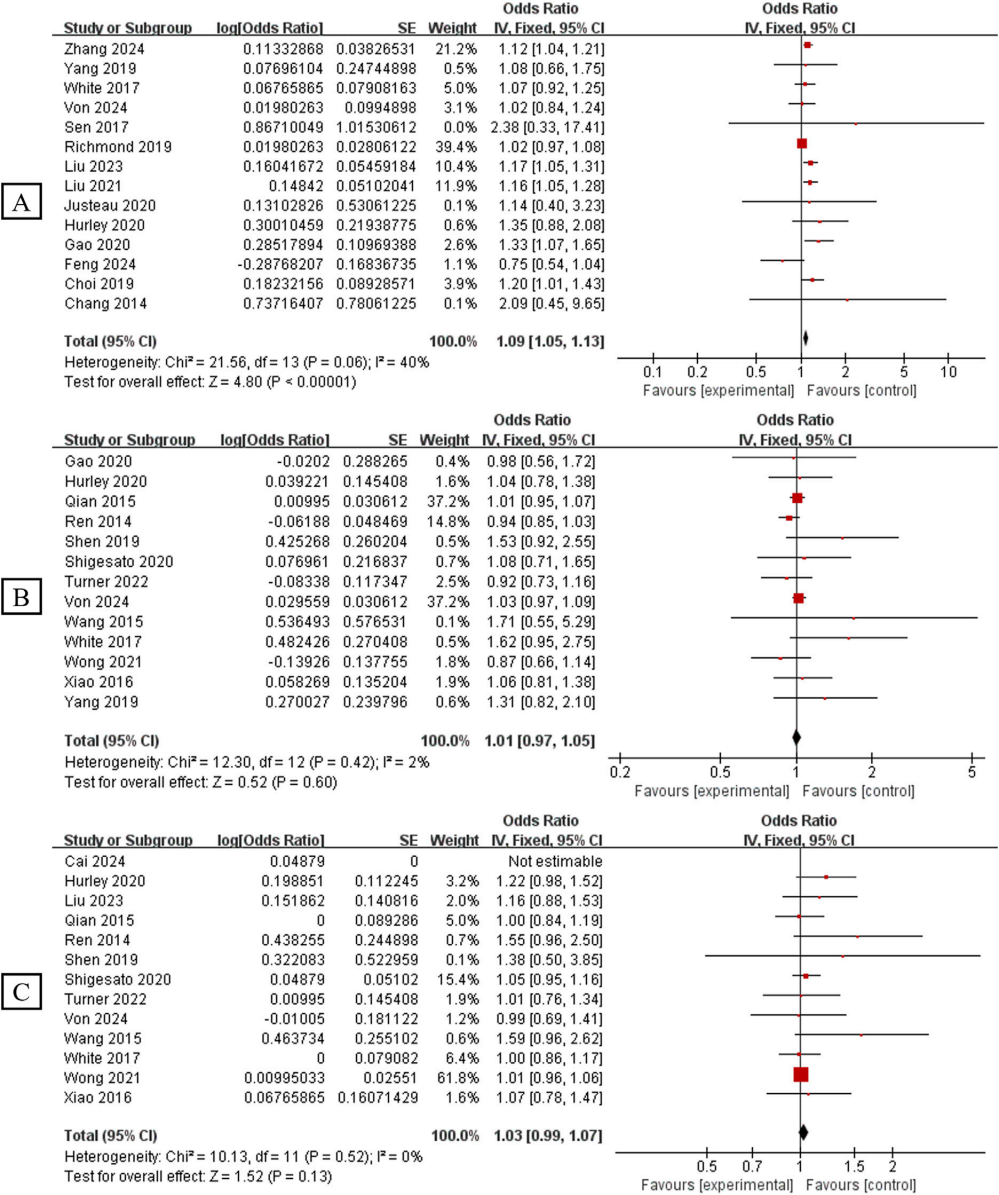
Association between sleep traits and risk of breast cancer incidence. **(A)**: Association between low quality sleep and risk of breast cancer incidence; **(B)**: Association between sleep duration <6 h and risk of breast cancer incidence; **(C)**: Association between sleep duration >9 h and risk of breast cancer incidence (Global, 2025).

Figure 2 shows different subgroup analyses of the association between sleep traits and breast cancer incidence risk. Fourteen studies were pooled for the subgroup where the sleep trait was estimation of low quality sleep (N = 1,507,688). The result indicated that low quality sleep increased breast cancer incidence risk (OR = 1.09, 95% CI 1.05–1.13, p < 0.00001, *I* ^2^ = 40%). Thirteen studies (N = 1,022,423) found no significant effect of sleepiness <6 h on breast cancer risk (OR = 1.01, 95% CI 0.97–1.05, p = 0.60, *I* ^2^ = 2%). Likewise, 13 studies (N = 1,084,028) found that drowsiness, quantified as sleep duration >9 h, did not have a significant effect on breast cancer risk (OR = 1.03, 95% CI 0.99–1.07, p = 0.13, *I* ^2^ = 0%) (Figure 2).

### Analysis of Sleep Traits Effects on Breast Cancer Adverse Progression and Mortality

**FIGURE 3:**
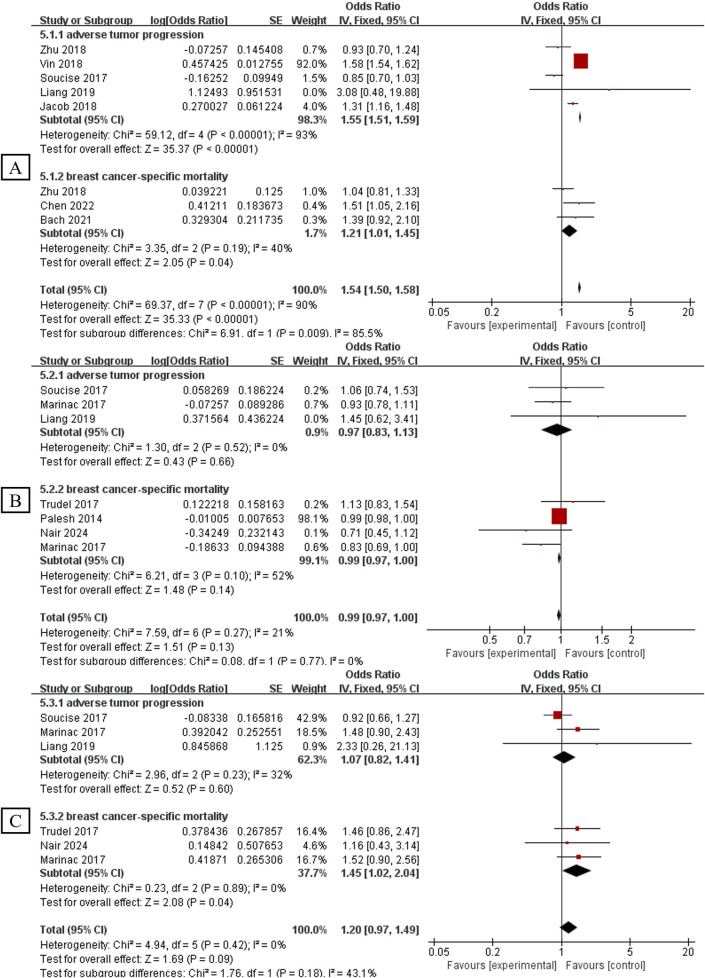
Association between sleep traits and risk of breast cancer progression (adverse progression and mortality). **(A)**: Association between low quality sleep and risk of breast cancer progression; **(B)**: Association between sleep duration <6 h and risk of breast cancer progression; **(C)**: Association between sleep duration >9 h and risk of breast cancer progression (Global, 2025).

Figure 3 shows different subgroup analyses of the association between sleep traits and breast cancer progression (adverse progression and mortality). In the analysis of progression risk, seven studies (N = 115,428) found that low quality sleep significantly increased the risk of breast cancer progression (OR = 1.54, 95%CI 1.50–1.58, p < 0.00001, *I* ^2^ = 90%). Six studies examining sleep duration <6 h (N = 13,394) showed no significant effect on the risk of breast cancer progression (OR = 0.99, 95%CI 0.97–1.00, p = 0.13, *I* ^2^ = 21%). Similarly, five studies focusing on sleep duration >9 h (N = 13,297) found that hypersomnia increased the risk of breast cancer progression (OR = 1.20, 95%CI 0.97–1.49, p = 0.09, *I* ^2^ = 0%) (Figure 3).

In the analysis of subgroup of adverse progression, five studiesresearches (N = 105,806) pooled indicated that low quality sleep increased the risk of breast cancer adverse progression (OR = 1.55, 95%CI 1.51–1.59, p < 0.00001, *I* ^2^ = 93%). However, sleep duration showed nonsignificant effect on the risk of breast cancer adverse progression, with three studies (N = 8,798) estimating sleep duration <6 h (OR = 0.97, 95%CI 0.83–1.13, p = 0.66, *I* ^2^ = 0%) and three studies (N = 8,798) estimating sleep duration >9 h (OR = 1.07, 95%CI 0.82–1.00, p = 0.60, *I* ^2^ = 32%) (Figure 3).

In the analysis of subgroup of breast cancer-specific mortality, three studies (N = 13,841) pooled indicated that low quality sleep increased breast cancer-specific mortality (OR = 1.21, 95%CI 1.01–1.45, p = 0.04, *I* ^2^ = 40%). Furthermore, the combined results of 4 studies (N = 7,643) showed nonsignificant outcome for sleep duration <6 h (OR = 1.21, 95%CI 1.01–1.45, p = 0.04, *I* ^2^ = 40%). On the contrary, pooled results from three studies found that sleep duration >9 h (N = 7,546) could increase breast cancer-specific mortality (OR = 1.45, 95%CI 1.02–2.04, p = 0.04, *I* ^2^ = 0%) (Figure 3).

## Discussion

With this systematic review, we aimed to assess the association of sleep quality and sleep duration with the overall disease process in breast cancer. The meta-analysis included 34 eligible studies to verify the effects of low sleep quality, short sleep duration, and prolonged sleep duration on breast cancer incidence, adverse progression, and specific mortality, respectively.

### Effects of Sleep Quality

Low quality sleep significantly increases the incidence and mortality risk of breast cancer, further validating those circadian rhythms, melatonin significantly influences tumor susceptibility and progression. For the occurrence of breast cancer, since the quality of sleep is closely related to the body’s immune system function, inflammatory response, hormone levels, lifestyle, etc., and low quality of sleep itself has been proven to be positively correlated with the long-term risk of various types of cancers, the result that low quality of sleep increases the incidence of breast cancer is to be expected [73, 74]. However, it has to be emphasized that although the effect of sleep quality on breast cancer incidence is significant, the estimated effect sizes are relatively small and do not elucidate the strong evidence for sleep quality on breast cancer risk.

For the progression of breast cancer, insufficient levels of melatonin secreted from the pineal gland in the dark may play a role in the association between sleep and breast cancer aggressiveness [68]. Low sleep quality, while not entirely representative of nighttime light and melatonin levels, may be directly related to melatonin levels, so melatonin can be listed as a possible key cause. There’s also the claim that sleep fragmentation leads to increased inflammatory cytokine production and natural killer cell dysfunction, potentially compromising immune surveillance against cancer progression [75]. This immune dysregulation creates a microenvironment conducive to tumor growth and metastatic spread. The clinical significance of these mechanisms is evident in prospective studies, where poor sleep efficiency (below 85%) has been associated with dramatically shorter survival times in women with advanced breast cancer (33.2 months versus 68.9 months) [31]. At the same time, studies have also pointed out that low quality of sleep can trigger negative attitudes towards death [76] and emotions such as depression and anxiety [77], which are detrimental to the health of breast cancer patients. The effect value of sleep quality on the progression impact of breast cancer is relatively large compared to the risk of morbidity. This suggests that we should pay more attention to sleep quality in breast cancer patients and emphasize the importance of sleep quality in the health management of this population.

From a clinical perspective, these findings support incorporating routine sleep quality assessments into oncology practice and providing targeted interventions such as cognitive behavioral therapy for insomnia to potentially improve patient prognosis [78].

### Effects of Sleep Duration

The available evidence suggests that sleep duration has no significant effect on breast cancer incidence and only suggests that sleep duration >9 h has a detrimental effect on the risk of breast cancer-specific mortality. Studies have shown that the metastatic spread of breast cancer is accelerated during sleep [79], that this metastatic spread occurs via circulating tumor cells (CTCs), and that resting periods are highly susceptible to metastasis, which may provide a rationale for the high rate of breast cancer-specific mortality associated with prolonged sleep. At the same time, the melatonin hypothesis, which suggests that shorter sleep is associated with lower melatonin levels, and the fact that melatonin is known to modulate susceptibility to cancer and has antiproliferative activity, suggests that the link between longer sleep and breast cancer is biologically plausible [80]. Some scholars have also suggested that excessive sleep may lead to elevated levels of systemic inflammation and an increase in some inflammatory biomarkers, such as CRP and IL-6, which may predispose individuals to breast cancer [81]. In fact, breast cancer patients, the subjects in whom breast cancer-specific deaths occur, due to the side effects of cancer treatment [82], are often accompanied by an increase in cortical activity related to arousal and a decrease in active cortex related to sleep homeostasis [16], so that sleep duration is more likely to be shorter, and prolonged sleep is an anomalous event in itself in this group. At the same time, it is important to note that the effect of prolonged sleep on breast cancer-specific mortality, while significant, only included three studies and did not provide strong evidence.

Overall, there is still controversy about the effect of sleep duration on breast cancer risk. Findings from a large multi-ethnic cohort study [60] suggest that both short and long sleep are associated with a higher risk of breast cancer incidence compared to normal sleep. According to the results of the Million Women Study [63], the overall prospective evidence does not support an association between sleep duration and breast cancer incidence risk. We consider that there is still a paucity of high-quality studies on sleep duration and the disease process in breast cancer, making it difficult to draw uniform conclusions. Therefore, more research is needed to validate the effects of short and long sleep duration on breast cancer development and progression, pinpointing the exact biological processes also remains elusive, necessitating additional investigation. Future studies should prioritize prospective designs with objective sleep measurements (e.g., actigraphy or polysomnography) to minimize recall bias [83], while incorporating molecular biomarkers such as circulating inflammatory cytokines, melatonin metabolites, and circadian gene expression profiles to elucidate whether the U-shaped association reflects direct causal mechanisms or underlying health conditions [84, 85]. Additionally, intervention studies examining whether sleep optimization through behavioral or pharmacological approaches can modify cancer trajectories would provide critical evidence for establishing sleep duration as a targetable risk factor in breast cancer prevention and management strategies [75].

Nevertheless, clinicians should be aware that excessive sleep duration (>9 h) may warrant further evaluation for underlying conditions such as depression, excessive fatigue, or disease progression that could contribute to poor outcomes [86].

### Limitations

Our study has several limitations. First, this study only analyzed the effects of sleep quality and sleep duration on breast cancer, and did not consider the effects of other sleep traits, such as daytime naps, nighttime lights, sleep preference type, nighttime awakenings, and sleep medication use, due to data limitations. Second, most of the studies focused on two regions, China and the United States, which may affect the representativeness of the findings on a global scale. Third, we included only English-language publications, which may have biased our findings. By including only studies published in English, we may have missed important local studies that are more likely to be published in journals other than English. Finally, a limitation that we cannot ignore is that due to differences in sleep quality measurement tools, heterogeneity in assessment tools (e.g., Pittsburgh Sleep Quality Index, polysomnography, self-reported measures) may affect the comparability of results and may affect the strength of observed associations.

### Conclusion

The available evidence points to sleep traits as primarily influencing progression in breast cancer patients and having a relatively small effect on breast cancer incidence. It can be confirmed that low quality sleep significantly increases adverse progression in breast cancer patients, suggesting that we should be concerned aboutsleep quality in breast cancer patients. Prolonged sleep may lead to breast cancer-specific mortality, but more research is needed in the future to continue to explore the impact of sleep duration and breast cancer risk.

## Abbreviations

OR, odds ratio; HR, hazard ratio; RR, relative risk; 95% CI, the 95% confidence interval; BC, breast cancer; SA, sleep apnea.
