# Supplementary material for: Sleep Traits to the Risk of Breast Cancer Disease Incidence, Adverse Progression and Mortality: Evidence From a Global Systematic Review and Meta-Analysis
Source: Int J Public Health. 2025 Jul 15;70:1608535. doi: 10.3389/ijph.2025.1608535 (PMC12303857; doi:10.3389/ijph.2025.1608535)
Supplement: Supplementary file 1 [file DataSheet1.pdf]

Supplementary Materials

Sleep traits to the risk of breast cancer disease process: Evidence from a global systematic review and meta-analysis

Contents

Search strategy for each database. 1

Table S1 Database 1: PubMed (Global, 2025). 1

Table S2 Database 2: Embase/ Medline (Global, 2025)... 2

Table S3 Database 3: Web of science (Global, 2025). 3

Table S4 Database 3: Cochrane library (Global, 2025). 4

Table S5 Risk of bias assessments according to Newcastle-Ottawa Scale (Global, 2025). 5

Table S6 Summarize of studies on breast cancer incidence and progression (Global, 2025)... 6

Figure S1 Funnel plot of included publications (Global, 2025). 16

Search strategy for each database.

Keyword: sleep; breast cancer; incidence/ adverse tumor progression/ worsening/ death/ mortality

Publication date: 2014-2024

Table S1 Database 1: PubMed (Global, 2025).

No.	Query	Items found
#1	(sleep [MeSH Terms]) OR (sleep [Title/Abstract])	263,160
#2	(breast cancer [MeSH Terms]) OR (breast cancer [Title/Abstract])	481,020
#3	(breast neoplasms [MeSH Terms]) OR (breast neoplasms [Title/Abstract])	367,657
#4	(breast cancer [MeSH Terms]) OR (breast cancer [Title/Abstract]) OR (breast neoplasms [MeSH Terms]) OR (breast neoplasms [Title/Abstract])	481,481
#5	(incidence [MeSH Terms]) OR (incidence [Title/Abstract])	1,123,860
#6	(adverse tumor progression [MeSH Terms]) OR (adverse tumor progression [Title/Abstract])	14,333
#7	(worsening [MeSH Terms]) OR (worsening [Title/Abstract])	75,263
#8	(death MeSH Terms]) OR (death [Title/Abstract])	971,328
#9	(mortality MeSH Terms]) OR (mortality [Title/Abstract])	1,107,550
#10	(incidence [MeSH Terms]) OR (incidence [Title/Abstract]) OR (adverse tumor progression [MeSH Terms]) OR (adverse tumor progression [Title/Abstract]) OR (worsening [MeSH Terms]) OR (worsening [Title/Abstract]) OR (death MeSH Terms]) OR (death [Title/Abstract]) OR (mortality MeSH Terms]) OR (mortality [Title/Abstract])	2,865,907
#11	((sleep [MeSH Terms]) OR (sleep [Title/Abstract])) AND ((breast cancer [MeSH Terms]) OR (breast cancer [Title/Abstract]) OR (breast neoplasms [MeSH Terms]) OR (breast neoplasms [Title/Abstract])) AND ((incidence [MeSH Terms]) OR (incidence [Title/Abstract]) OR (adverse tumor progression [MeSH Terms]) OR (adverse tumor progression [Title/Abstract]) OR (worsening [MeSH Terms]) OR (worsening [Title/Abstract]) OR (death MeSH Terms]) OR (death [Title/Abstract]) OR (mortality MeSH Terms]) OR (mortality [Title/Abstract]))	266
#12	Studies with publication date from 2014 to 2024 are included.	197

Table S2 Database 2: Embase/ Medline (Global, 2025).

No.	Query	Results
#1	(' sleep ':ti,ab,kw OR ' sleep '/exp) AND ([embase]/lim OR [medline]/lim) AND [2014-2024]/py	332,990
#2	(' breast cancer ':ti,ab,kw OR ' breast cancer '/exp) AND ([embase]/lim OR [medline]/lim) AND [2014-2024]/py	397,914
#3	(' breast neoplasms ':ti,ab,kw OR ' breast neoplasms '/exp) AND ([embase]/lim OR [medline]/lim) AND [2014-2024]/py	374,608
#4	#2 OR #3	411,380
#5	('incidence':ti,ab,kw OR 'incidence'/exp) AND ([embase]/lim OR [medline]/lim) AND [2014-2024]/py	906,939
#6	('adverse tumor progression':ti,ab,kw OR 'adverse tumor progression'/exp) AND ([embase]/lim OR [medline]/lim) AND [2014-2024]/py	0
#7	('worsening':ti,ab,kw OR 'worsening'/exp) AND ([embase]/lim OR [medline]/lim) AND [2014-2024]/py	114,698
#8	('death':ti,ab,kw OR 'death'/exp) AND ([embase]/lim OR [medline]/lim) AND [2014-2024]/py	1,633,543
#9	('mortality':ti,ab,kw OR 'mortality'/exp) AND ([embase]/lim OR [medline]/lim) AND [2014-2024]/py	1,245,198
#10	#5 OR #6 OR #7 OR #8 OR #9	2,687,783
#11	#1 AND #4 AND #10	862

Table S3 Database 3: Web of science (Global, 2025).

No.	Query	Results
#1	TS=sleep	781,777
#2	TS= breast cancer	1,167,871
#3	TS= breast neoplasms	881,386
#4	TS= breast cancer OR TS= breast neoplasms	1,260,513
#5	TS= incidence	1,989,433
#6	TS= adverse tumor progression	40,966
#7	TS= worsening	227,089
#8	TS=death	2,446,096
#9	TS= mortality	2,850,076
#10	TS= incidence OR TS= adverse tumor progression OR TS= worsening OR TS=death OR TS= mortality	6,281,722
#11	(TS=sleep) AND (TS= breast cancer OR TS= breast neoplasms) AND (TS= incidence OR TS= adverse tumor progression OR TS= worsening OR TS=death OR TS= mortality)	2,764
#12	Studies with publication date from 2014 to 2024 are included.	2,156

Table S4 Database 3: Cochrane library (Global, 2025).

No.	Query	Results
#1	sleep in Title Abstract Keyword	153
#2	breast caner in Title Abstract Keyword	1
#3	breast neoplasms in Title Abstract Keyword	74
#4	#2 OR #3	74
#5	incidence in Title Abstract Keyword	564
#6	adverse tumor progression in Title Abstract Keyword	127
#7	worsening in Title Abstract Keyword	158
#8	death in Title Abstract Keyword	547
#9	mortality in Title Abstract Keyword	507
#10	#5 OR #6 OR #7 OR #8 OR #9	835
#11	#1 AND #4 AND #10	15
#12	Studies with publication date from 2014 to 2024 are included.	10

Table S5 Risk of bias assessments according to Newcastle-Ottawa Scale (Global, 2025).

Reference	Study Type	Selection	Comparability	Exposure/ Outcome	Total
Zhang et al. (2024)	Cohort	****	**	***	9
Yang et al. (2019)	Case control	****	**	**	8
White et al. (2017)	Cohort	****	**	***	9
Von et al. (2024)	Cohort	***	**	***	8
Sen et al. (2017)	Cohort	****	**	***	9
Richmond et al. (2019)	Case control	****	**	***	9
Liu et al. (2023)	Case control	***	**	**	7
Liu et al. (2021)	Cohort	****	**	***	9
Justeau et al. (2020)	Cohort	****	**	***	9
Hurley et al. (2020)	Case control	****	**	***	9
Gao et al. (2020)	Case control	***	**	**	7
Feng et al. (2024)	Case control	***	**	**	7
Choi et al. (2019)	Cohort	****	**	***	9
Chang et al. (2014)	Cohort	****	**	***	9
Qian et al. (2015)	Cohort	****	**	**	8
Ren et al. (2014)	Case control	**	*	***	6
Shen et al. (2019)	Cohort	****	**	***	9
Shigesato et al. (2020)	Cohort	****	**	***	9
Turner et al. (2022)	Case control	*	*	***	5
Wang et al. (2015)	Case control	****	*	***	8
Wong et al. (2021)	Cohort	****	**	***	9
Xiao et al. (2016)	Case control	****	**	***	9
Cai et al. (2024)	Case control	****	**	***	9
Zhu et al. (2018)	Cohort	****	-	***	7
Vin et al. (2018)	Cohort	****	**	**	8
Soucise et al. (2017)	Cohort	****	**	***	9
Liang et al. (2019)	Cohort	****	**	***	9
Jacob et al. (2018)	Case control	****	**	***	9
Chen et al. (2022)	Cohort	****	**	***	9
Bach et al. (2021)	Cohort	****	**	***	9
Marinac et al. (2017)	Cohort	****	**	***	9
Trudel et al. (2017)	Cohort	***	**	***	8
Palesh et al. (2013)	Cohort	***	**	***	8
Nair et al. (2024)	Cohort	****	**	**	8

Table S6 Summarize of studies on breast cancer incidence and progression (Global, 2025)..

Author/Year	Sample source	Sleep assessment	Breast cancer risk assessment	Confounding factors adjusted	Main conclusion
Zhang et. al. 2024	Participants in UK Biobank excluded cancer patients, miss information and skin cancer patients during follow-up	Sleep score (five self-reported sleep and circadian behaviors, including duration, daytime napping, chronotype, insomnia symptom, and snoring.generated a weight sleep score by using the equation)	Age-standardized incidence of site-specific cancer	Age at recruitment, sex, ethnicity, education, TDI, alcohol intake, height, body mass index, use of NSAIDs, family history of cancer, diabetes, hypertension, CVD, age at menarche, number of live birth, use of hormones and use of oral contraceptive, colorectal cancer screening	Low TPA-poor sleep group and high SB-poor sleep group had the highest risk for overall cancer, breast cancer, and lung cancer.
Yang et. al. 2019	Patients diagnosed with breast cancer in medical charts in study JBCS, match with her neibours	Sleep quality(duration, daytime napping, chronotype, insomnia symptom, and snoring)<br>Sleep duration	Breast cancer incidence	Age, education, family income, occupation, marital status, height, body weight, age at menarche, age at first birth, menopausal status, oral contraceptive, the number of live births, regular physical activity, cigarette smoking, alcohol drinking, tea drinking, family history of BC, and use of menopausal hormones	Sleep problems including light exposure at night, night/shift work, late sleeping, and frequent night waking could increase the risk of BC development, independent of other sleep factors.
White et. al. 2017	Woman having a sister who had been diagnosed with breast cancer from the	Difficulty sleeping on a regular basis<br>Sleep duration	Incident breast cancer cases	Demographics (race/ethnicity, education, income and marital status), reproductive history, lifestyle factors such as smoking and alcohol consumption and use of exogenous hormones was obtained. Menopausal	Most sleep characteristics, including sleep duration, were not associated with an increased risk although

Author/Year	Sample source	Sleep assessment	Breast cancer risk assessment	Confounding factors adjusted	Main conclusion
	Sister Study Data Release 5.01.			status (premenopausal vs. Ost menopausal); body mass index	higher risk was observed for some markers of inadequate or poor-quality sleep.
Von et. al. 2024	Active and retired females enrolled in California's State Teachers Retirement System	Sleep quality (a modified version of the GSI,overall sleep quality, latency (how long it takes to fall asleep), duration (hours per night), disturbance (trouble falling asleep, waking in the night, or waking too early), and sleep medication use)	Breast cancer diagnosis	Age, race/ ethnicity, family history of breast cancer, body mass index (BMI), physical activity, marital status, age at menopause, use of hormone replacement therapy, use of pain medication or onsteroidal anti-inflammatory drugs, comorbidities reported at Q5 (diabetes, depression, chronic obstructive pulmonary disease, Parkinson's disease, chronic fatigue syndrome, lupus, irritable bowel syndrome, Crohn's disease, and multiple sclerosis), household income, education level, smoking history, alcohol consumption, age at menarche, pregnancy history, and breast feeding history	Measures of sleep quality did not appear to be associated with subsequent breast cancer risk. The HR for evening chronotypes compared to morning chronotypes was somewhat elevated
Sen et. al. 2017	Residents in Nord-Trøndelag Health Study (HUNT study)	Have all insomnia symptoms simultaneously	Breast cancer incidence	Age at first birth and parity, physical activity, alcohol consumption, smoking status, level of education, and shift work, BMI, psychological symptoms of anxiety and depression, use of sleep medication/sedatives	Our results suggest that having only some aspects of insomnia may not predispose someone to breast cancer. In contrast, experiencing all insomnia symptoms simultaneously might confer considerable excess risk.

Author/Year	Sample source	Sleep assessment	Breast cancer risk assessment	Confounding factors adjusted	Main conclusion
Richmond et. al. 2019	Woman recruited by UK biobank	Insomnia symptoms	Breast cancer incidence	Education, body mass index (BMI), alcohol intake, smoking, strenuous physical activity, family history of breast cancer, age at menarche, parity, use of oral Contraceptives, menopause status, and hormone replacement therapy	Consistent evidence for a protective effect of morning preference and suggestive evidence for an adverse effect of increased sleep duration on breast cancer risk
Liu et. al. 2023	Woman residents from the breast cancer cohort study in Chinese women (BCCSCW)	Sleep satisfaction; Sleep duration	Breast cancer incidence	Age, education, marital status, annual family income, and history of benign breast disease	limited sleep duration, and reduced sleep satisfaction could all be considered risk factors for the development of breast cancer
Liu et. al. 2021	data from the Taiwan National Health Insurance Research Database.	Insomnia	Incidence rate of breast cancer	Age, insured amount, urbanization, and residential location; related comorbidities; sleeping pills	women with insomnia had increased risk of breast cancer, especially those in high urbanization or with high insured amounts.
Justeau et. al. 2020	Patients from the French administrative health care database (SNDS)	Nocturnal hypoxemia	Breast cancer incidence	Age, gender, body mass index, smoking status, alcohol intake, diabetes, hypertension, medical history of cardiac and chronic obstructive pulmonary disease, marital status, type of sleep study, and study site	Nocturnal hypoxemia was associated with all-cancer incidence in patients investigated for OSA.

Author/Year	Sample source	Sleep assessment	Breast cancer risk assessment	Confounding factors adjusted	Main conclusion
Hurley et. al. 2020	Active and retired teachers form California Teachers Study (CTS).	Pittsburg Sleep Quality Index	Breast cancer incidence	Age, race (white/non-white), total pack-years of smoking, age at first full-term pregnancy, BMI, physical activity, family history of breast cancer, age at menopause, medication use for depression, NSAID use, and marital status	Increased breast cancer risks were associated with sleep deficiency. With the exception of duration, linear increases in risk were Associated with all the other individual components of sleep deficiency
Gao et. al. 2020	Patients visited in the Second Hospital of Shanxi Medical University	Obstructive sleep apnea syndrome	Breast cancer incidence	Age, smoking status, family history of cancer and BMI	Study found a detrimental causal effect of OSAS on BC risk
Feng et. al. 2024	data from the UK Biobank	Sleeplessness	Breast cancer incidence	Body mass index, waist circumference, age at menarche, birth weight, physical activity, alcohol consumption, smoking habits, and diabetes	Sleeplessness does not exhibit a statistically significant relationship with either overall breast cancer or its subtypes
Choi et. al. 2019	Outpatient and inpatient data from KNHIS	Obstructive sleep apnea	Breast cancer incidence	Age, sex, body mass index, and smoking	OSA may be a risk factor for breast cancer in women.
Chang et. al. 2014	Patients data from Longitudinal Health Insurance Database (LHID) 2005.	Sleep apnea	Breast cancer incidence	Age, monthly income, urbanization level, geographic region, hypertension, hyperlipidemia, diabetes, alcohol use disorder, and obesity	The findings of our population-based study suggest an association between SA and an

Author/Year	Sample source	Sleep assessment	Breast cancer risk assessment	Confounding factors adjusted	Main conclusion
					increased risk of breast cancer in women
Qian et. al. 2015	Woman data from the BCDDP study.	Sleep duration	Breast cancer incidence	Age, race, education, marital status, body mass index, vigorous physical activity, smoking status, pack-year, year since quitting, age at first live birth, number of live birth, age at first birth, menopause, use of hormonal replacement therapy, use of multivitamin, history of diabetes, family history of cancer and alcohol consumption	We found no association between sleep and overall Breast cancer. However, we observed a decreased risk of ER þ PR þ breast cancer (RR o6 vs 8 – 9 h (95% CI): 0.54 (0.31, 0.93), P for trend, 0.003) with shorter sleep duration
Ren et. al. 2014	Patients with incident invasive BC and age-matched female controls	Sleep duration	Breast cancer incidence	Age, education, BMI, marital status, age at menarche, menopausal status, parity, activity, breastfeeding and family history of breast cancer	Abnormal sleep increases BC risk and longer sleep duration may not compensate the effect of night shift work on risk of BC
Shen et. al. 2019	Mexican American adults identified from Mano-A-Mano, an Mexican American cohort study and follow up	Sleep duration	Breast cancer incidence	Birthplace, language acculturation, age, sex, marital status, education level, smoking status, drinking status, sitting time, physical activity, and BMI category	Sleeping less than 6 hours per night and at least 9 hours per night increased the risk of breast cancer
Shigesato et. al. 2020	Participants in the multiethnic cohort (MEC)	Sleep duration	Breast cancer incidence	BMI, ethnicity, age, education, family history of breast cancer, smoking status, alcohol use, physical activity,	Findings provide little support for a role of sleep duration as

Author/Year	Sample source	Sleep assessment	Breast cancer risk assessment	Confounding factors adjusted	Main conclusion
	include whites, Native Hawaiians and Japanese Americans recruited in Hawaii and African Americans and Latinos recruited in California, primarily Los Angeles.			age at menarche, age at first live birth, number of children, hormone treatment, menopausal status, caffeine intake and total energy intake	reported in middle age in breast cancer development
Turner et. al. 2022	Patients data from MCC-Spain study and control data matched from public primary health centres	Sleep duration	Breast cancer incidence	Age, centre, education, socioeconomic status, cigarette smoking status, family history of prostate cancer in first degree relatives, BMI, physical activity, alcohol consumption. Categories for missing values were created for family history of prostate cancer in first degree relatives and alcohol consumption	There was no clear association between various sleep characteristics and breast or prostate cancer risk observed
Wang et. al. 2015	Female patients with recent histologically diagnosed primary breast cancer	Sleep duration	Breast cancer incidence	Age, education, BMI, age at menarche, menopausal status, parity, physical activity, breast-feeding, family history of breast cancer, and other sleep factors (24-h sleep duration, night-shift work, or daytime napping)	Sleep problems, including night-shift work, and shorter and longer sleep duration, are associated with an increased breast cancer risk
Wong et. al. 2021	Woman recruited through the UK Breast Cancer Screening Programme in the Million Women Study	Sleep duration	Breast cancer risk	Region of residence, educational attainment, Townsend deprivation index, strenuous exercise, age at menarche, height, age at first birth, and parity; alcohol intake	The totality of the prospective evidence does not support an association between sleep duration and breast cancer risk.

Author/Year	Sample source	Sleep assessment	Breast cancer risk assessment	Confounding factors adjusted	Main conclusion
Xiao et. al. 2016	Participants from The SCCS study	Sleep duration	Breast cancer incidence	Age, enrollment year, enrollment state, race, education, income, marital status, body-mass index, moderate-to-vigorous physical activity, overall sitting, smoking status, pack-year, number of live birth, age at first birth, length of breast feeding, age at menarche, menopause, use of menopausal hormone therapy, use of multivitamin, use of aspirin, history of diabetes, family history of cancer, alcohol consumption, and dietary intakes of total fat, fiber, folate and total calories	Short sleep duration may be a risk factor for hormone receptor-negative breast cancer among black women
Cai et. al. 2024	Resisent data were extracted from 5 independent cross-sectional waves of NhaNes	Sleep duration	Breast cancer incidence	Age, education, race, PIR, BMI, Marital status, hypertension and diabetes, smoking and alcohol consumption	No significant association was observed between sleep duration and breast cancer
Zhu(2018)	Women diagnosed with stage I-III breast cancer in the Shanghai Breast Cancer Survival Study.	Sleep difficulty and low sleep quality	Breast cancer recurrence and specific mortality	-	We did not observe any significant association of sleep difficulty and poor sleep quality with increased risk of death and recurrence in breast cancer patients
Vin(2018)	Patients data from the Healthcare Cost and Utilization Project (HCUP) Nationwide Inpatient	sleep disorder (insomnia, sleep-related breathing disorders, hypersomnia, circadian rhythm sleep	Complications	Age, race, stage, income, insurance, residential region, year of discharge, comorbidities, and surgical treatment	There was no significant difference in the odds of in-hospital mortality among patients with a sleep

Author/Year	Sample source	Sleep assessment	Breast cancer risk assessment	Confounding factors adjusted	Main conclusion
	Sample (NIS)	disorder, parasomnia, sleep-related movement disorder, and other sleep disorders			disorder. But the odds of clinical complications were significantly higher
Soucise(2017)	Woman diagnosed primary invasive breast cancer.	Sleep quality sleep duration	Tumor stage	Age, BMI, and HT use, income, smoking status, pack years, alcohol intake, and physical activity	Aspects of sleep (sleep duration and quality), partially modifiable health behaviors, may be associated with development of aggressive tumor characteristics in postmenopausal women
Liang(2019)	Patients recruited in the Guangzhou Breast Cancer Study (GZBCS) in China	Sleep quality (Pittsburgh Sleep Quality Index) and daytime napping), duration	Breast cancer progression	Age at diagnosis, stage, chemotherapy, hormone therapy, HER2 status, scores of Charlson Comorbidity Index, BMI, menopausal status (pre-menopausal/post-menopausal) and educational level	Poor sleep quality, short and long sleep duration were associated with an increased risk of breast cancer progression, particularly for pre-menopausal women
Jacob(2018)	Patients diagnosed with breast cancer from nationwide Disease Analyzer database (IQVIA)	Sleep disorders	Breast cancer patients with metastases	Age, index year, physician, type of hormonal therapy (tamoxifen or aromatase inhibitors) and follow-up time, depression.	A positive association was found between sleep disorders and the presence of metastases in women diagnosed with breast cancer

Author/Year	Sample source	Sleep assessment	Breast cancer risk assessment	Confounding factors adjusted	Main conclusion
					in gynecological practices in Germany
Chen(2022)	baseline:Taiwan Cancer Registry Database (TCRD)	Pre-existing sleep disorders	Survival outcomes of women receiving standard treatments for breast invasive ductal carcinoma	Age, income level, urbanization, menopausal status, HER2 status, AJCC clinical stage, CCI score, type of breast surgery, and the presence of diabetes, hyperlipidemia, ESRD, liver cirrhosis, AMI, CAD, stroke, hormone receptors, differentiation, nodal surgery, chemotherapy, and adjuvant RT	The sleep disorder group had poorer survival rates than the non-sleep disorder group in breast cancer
Bach(2021)	Patients first diagnosed with breast cancer from the Disease Analyzer database (IQVIA)	Sleep disorders	Death within 5 years	Age, diabetes mellitus, obesity, lipid metabolism disorders, atrial hypertension, ischemic heart diseases, renal insufficiency, thyroid gland disorders, depression, anxiety disorder	Sleep disorders are significantly associated with an increased mortality rate
Marinac(2017)	Participants were breast cancer survivors from the WHEL Study	Sleep duration	Breast cancer recurrence breast cancer-specific mortality	Age, stage, grade, body mass index (kg/m2), number of co-morbidities, race/ethnicity, intervention group, and study site	Consistent long or short sleep, which may reflect inter-individual variability in the need for sleep, does not appear to influence prognosis among early-stage breast cancer survivors
Trudel(2017)	Baseline:Nurses diagnosed breast cancer from The Nurses' Health Study	Sleep duration	Breast cancer-specific mortality	Year of diagnosis, age at diagnosis, time since diagnosis, cancer stage, surgery, chemotherapy, radiation therapy, hormone therapy, prevalent diabetes or heart disease, missing indicators for oncologic	Sleep duration was associated with higher breast-cancer mortality risk

Author/Year	Sample source	Sleep assessment	Breast cancer risk assessment	Confounding factors adjusted	Main conclusion
				treatments, age, marital status, education level, income, OC use, number of pregnancies, family history of breast cancer, menopausal status, PMH use, BMI, alcohol consumption, smoking, caffeine, calories intake, physical activity,	
Palesh(2013)	Participants were recruited and followed-up. woman diagnosed breast cancer	Shorter time in bed	Predictive breast cancer mortality	Age, estrogen receptor status, treatment [chemotherapy, radiation therapy, hormonal treatment], dominant site of metastatic disease spread [viscera, bone, and chest wall], depression, and cortisol levels	Better sleep efficiency and less sleep disruption are significant independent prognostic factors in women with advanced breast cancer
Nair(2024)	Participants diagnosed with primary invasive breast cancer in The WEB Study	Short sleep<br>Long sleep	Mortality outcomes were ascertained using the National Death Index	Age, race/ethnicity, years of education, body mass index (BMI), menopausal status, pack-years of smoking, tumor stage, and estrogen-receptor (ER)	Sleep duration was not associated with either AC or BC mortality

Figure S1 Funnel plot of included publications (Global, 2025).

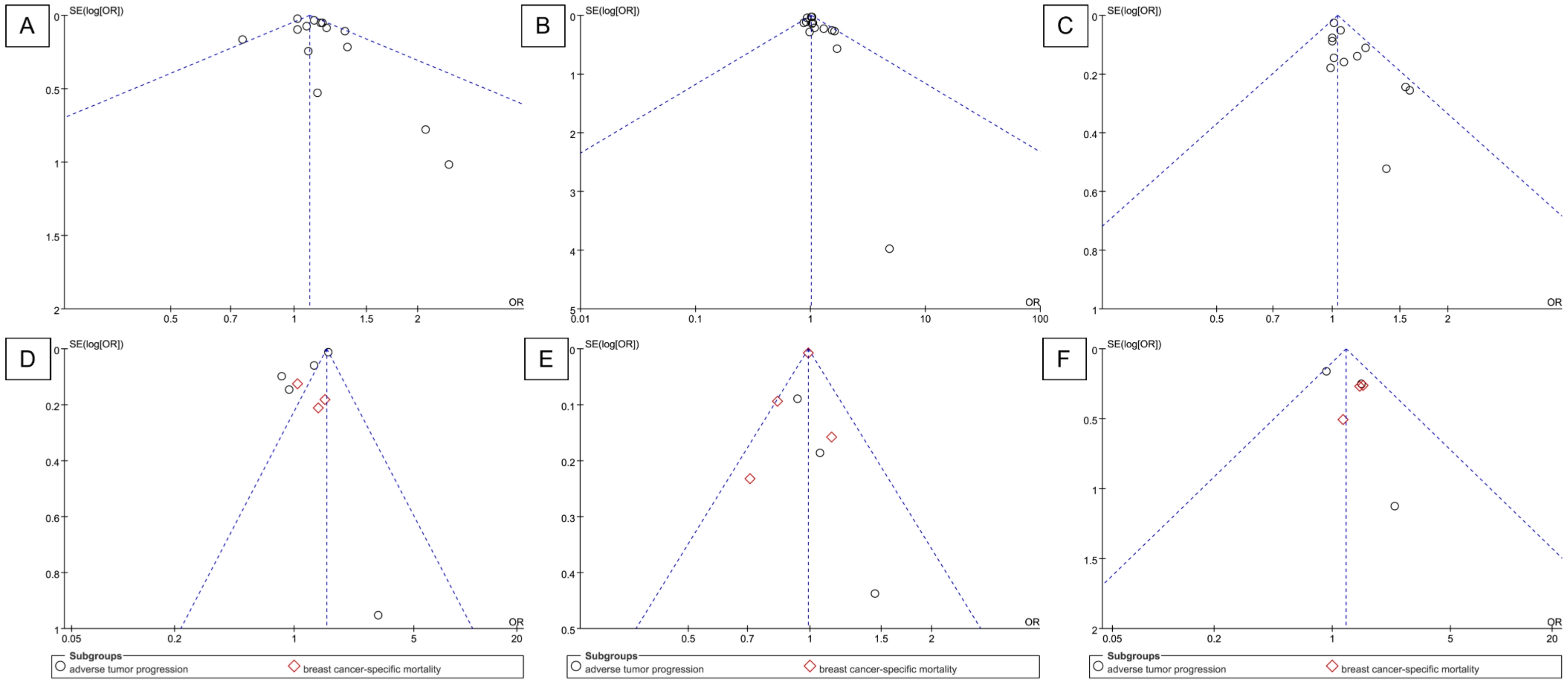

Note: A: publications of low quality sleep to breast cancer incidence; B: publications of sleep duration <6h to breast cancer incidence; C: publications of sleep duration >9h to breast cancer incidence; D: publications of low quality sleep to breast cancer progression; E: publications of sleep duration <6h to breast cancer progression; F: publications of sleep duration >9h to breast cancer progression.
